# Interplay between long‐term vulnerability and new risk: Young adolescent and maternal mental health immediately before and during the COVID‐19 pandemic

**DOI:** 10.1111/jcv2.12008

**Published:** 2021-05-18

**Authors:** Nicola Wright, Jonathan Hill, Helen Sharp, Andrew Pickles

**Affiliations:** ^1^ Department of Biostatistics & Health Informatics Institute of Psychiatry, Psychology & Neuroscience King's College London London UK; ^2^ School of Psychology and Clinical Language Sciences University of Reading Reading Berkshire UK; ^3^ Department of Life and Human Sciences University of Liverpool Liverpool Merseyside UK

**Keywords:** adolescence, behaviour problems, depression, longitudinal studies, sex differences

## Abstract

**Background:**

We examine whether there has been an increase in young adolescent and maternal mental health problems from pre‐ to post‐onset of the COVID‐19 pandemic.

**Methods:**

Children aged 11–12 years and their mothers participating in a UK population‐based birth cohort (Wirral Child Health and Development Study) provided mental health data between December 2019 and March 2020, and again 3 months after lockdown, 89% (*N* = 202) of 226 assessed pre‐COVID‐19. Emotional and behavioural problems were assessed by self‐ and maternal reports, and long‐term vulnerability by maternal report of prior child adjustment, and maternal prenatal depression.

**Results:**

The young adolescents reported a 44% (95% confidence interval [CI: 23%–65%]) increase in symptoms of depression and 26% (95% CI [12%–40%]) for post‐traumatic stress disorder, with corresponding maternal reports of child symptoms of 71% (95% CI [44%–99%]) and 43% (95% CI 29%–86%). Disruptive behaviour problem symptoms increased by 76% (95% CI [43%–109%]) particularly in children without previous externalising symptoms. Both female gender and having had high internalising symptoms earlier in childhood were associated with elevated rates of depression pre‐pandemic, and with greater absolute increases during COVID‐19. Mothers' own depression symptoms increased by 42% (95% CI [20%–65%]), and this change was greater among mothers who had prenatal depression. No change in anxiety was observed among children or mothers. None of these increases were moderated by COVID‐19‐related experiences such as frontline worker status of a parent. Prior to the pandemic, rates of maternal and child depression were greater in families experiencing higher deprivation, but changed only in less deprived families, raising their rates to those of the high deprivation group.

**Conclusions:**

COVID‐19 has led to a marked increase in mental health problems in young adolescents and their mothers with concomitant requirements for mental health services to have the resources to adapt to meet the level and nature of the needs.

## INTRODUCTION

There is widespread concern regarding the impact of the COVID‐19 pandemic and associated lockdown and social distancing measures on the mental health of children and adolescents. However, there is sparse and inconsistent evidence on whether exposure to the pandemic has been associated with a rise in mental health problems. Here we report on the mental health of 12‐year‐old children from a general population birth cohort, comparing their levels of emotional and behavioural problems immediately prior to, and during, the pandemic. This provides a design almost as strong as a randomised control trial, so the study findings can be readily attributed to a COVID‐19 pandemic effect at a crucial time point in the emergence of vulnerability for depression.

**Key points**

- Evidence is not yet available from well‐characterised samples on whether COVID‐19 has caused an increase in children's mental health problems.
- Utilising measurement immediately before and during the pandemic, we find COVID‐19 has increased depression in young adolescents, adding risk for those with long‐standing emotional problems and for girls at an age when they are vulnerable to developing depression. COVID‐19 disproportionately increased disruptive behaviour problems among children without prior difficulties.
- Referrals to clinical services during the pandemic are likely to comprise young people with new onsets of mental health problems for which COVID‐19‐related stressors may be the most relevant, and others where COVID‐19 has added to pre‐existing vulnerability with a need for attention to both.

Prospective studies, such as Co‐Space (Pearcey et al., 2020), with measurement starting soon after the lockdown are providing invaluable information on the time course of children's emotional and behavioural problems. Follow‐up has indicated increases of parent‐reported emotional and behavioural problems in children up to age 11 and a decrease of parent‐reported emotional problems in adolescents, with adolescent self‐report indicating no change in symptoms. In the recent report ‘NHS Mental Health in Children Study’ (Vizard, Sadler, et al., 2020), a post‐COVID follow‐up was collected on a sample of 5–22 years old (*N* = 3570, 45% follow‐up rate) who were assessed 3 years prior to the pandemic. The study reported an increase in rates of probable mental disorder as reported by parents in 5–16‐year olds. The increase and absolute rates were similar in boys and girls. However, pre‐ and post‐lockdown status was heavily confounded with time, and this limits the ability to attribute the increase to the pandemic. Two studies have provided a more direct test of an increase associated with the pandemic by comparing measurement prior to, and during, the pandemic. Based on parent, but not child, report, Bignardi et al. (2020) found an increase in depressive but not anxiety symptoms in children aged 7–11 years who had been assessed 1 year prior to the pandemic (*n* = 168, 31% follow‐up rate). With child, but not parent, report, Widnall et al. (2020) found no increase in depressive symptoms and a slight decrease in anxiety symptoms in 13–14‐year olds (*n* = 770, 44% follow‐up rate) who had been assessed 5 months prior to the pandemic.

Studies of young adults have provided more consistent evidence of a rise in depression in young women. A study of adults with assessment immediately pre‐pandemic (*n* = 3000) reported an increase in moderate and severe depression, with the greatest increase in women in the age range 16–39 years (Vizard, Davis, et al., 2020). A greater increase in mental health symptoms in younger women and parents, and amongst those with prior mental health problems, was reported based on pre‐pandemic assessments made several years earlier in one further study (*n* = 15,376, Pierce et al., 2020; *n* = 17,452, Banks & Xu, 2020).

In the context of the established gender difference in depression that emerges between ages 11 and 14, with levels rising more in girls than boys (Cyranowski et al., 2000), we examine whether any increase in young adolescent mental health symptoms is moderated by child gender. We also ask whether vulnerability to COVID‐19 reflects long‐term pre‐existing vulnerability (Clark et al., 2020), which we assessed in young adolescents as emotional and behavioural problems at age seven years, and in mothers as depression when they were pregnant with the study children. We further examine whether any increase in mental health problems is moderated by a range of factors identified in existing research or opinion pieces on the pandemic. This includes living in a deprived neighbourhood, inter‐partner abuse in the home and specific aspects of COVID‐19 exposure, such as having a frontline worker in the household, home‐schooling whilst home‐working or being exposed to COVID‐19 associated stressors, both financial and non‐financial (Banks & Xu, 2020; Clark et al., 2020; Pierce et al., 2020; Vizard, Davis, et al., 2020).

In this paper, we report findings of preregistered analyses comparing the mental health of young adolescents and their mothers assessed over the 3 months pre‐pandemic to that assessed over months June to August after the lockdown in the United Kingdom. We also examine whether there has been a rise in inter‐partner abuse reported by the mother. We examine differential effects on young adolescent mental health by gender and prior adjustment problems, and on mothers' mental health by depression when first recruited during pregnancy. We explore whether neighbourhood deprivation, inter‐partner abuse in the home and specific aspects of COVID‐19 exposure moderate any changes in mental health problems.

## METHOD

### Sample

The study is embedded in the Wirral Child Health and Development Study (WCHADS), a prospective epidemiological child development study of a sample of first‐time mothers (*n* = 1233) (see Sharp et al. [2012] for sampling description). There had been 11 waves of data collection up to age 9 years when 760 families provided data (from a pool of 812 families who have provided consent for ongoing follow‐up). Socio‐economic conditions on the Wirral range between the deprived inner city and affluent suburbs with very low numbers from ethnic minorities. The mean age at recruitment in pregnancy was 26.8 years (SD = 5.8, range 18–51), 41.8% of the sample were in the most deprived quintile of UK neighbourhoods (2003 Indices of Multiple Deprivation, IMD; Noble et al., 2004) and 96.1% were White British.

**FIGURE 1:**
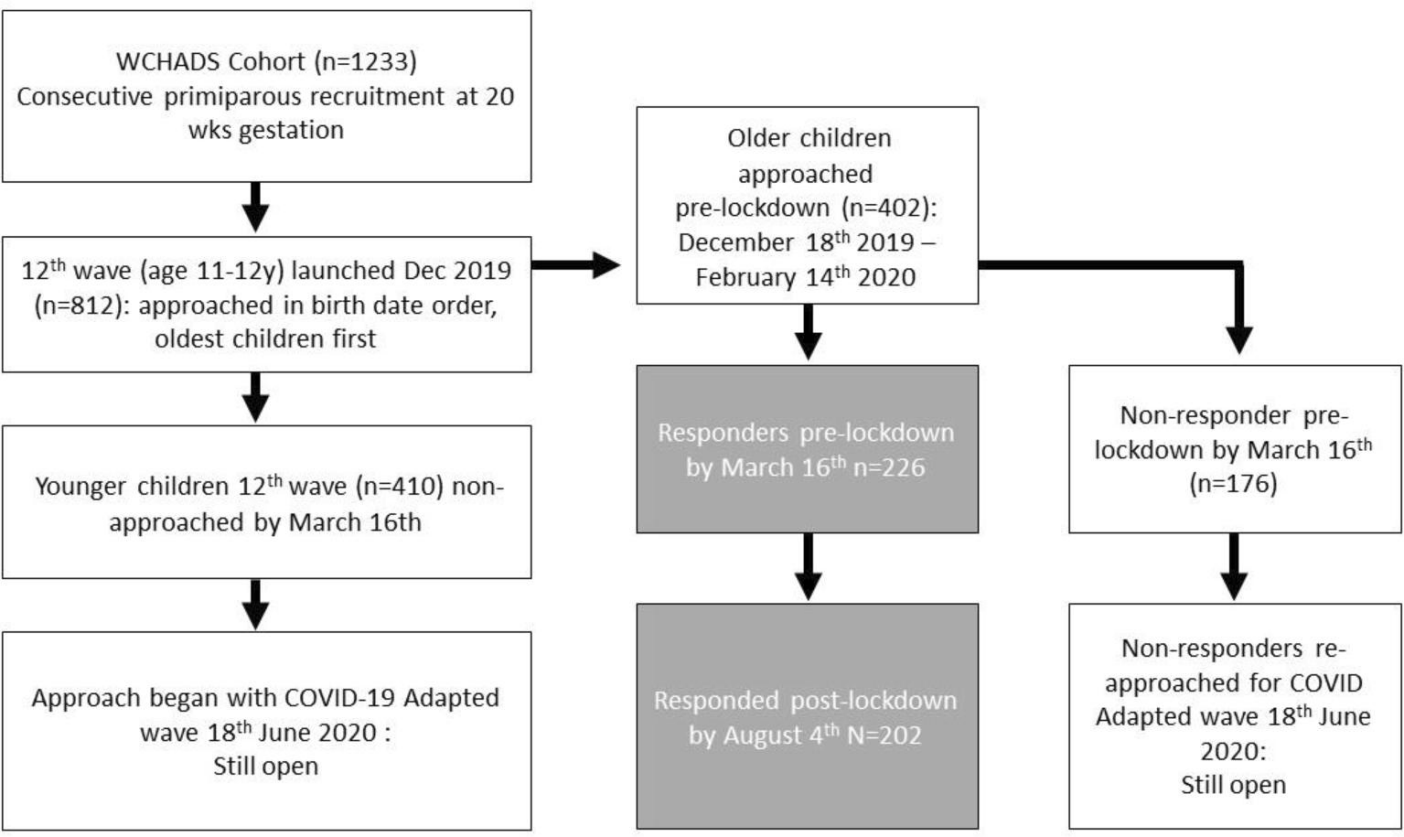
Wirral Child Health and Development Study (WCHADS) participant flow diagram from recruitment to post‐pandemic assessment

The sample for this study, shown in the grey boxes in Figure 1, comprised 226 families who had provided data for pre‐COVID‐19 during the 12th wave of the WCHADS up to the day after the UK social distancing measures were implemented on the 16^th^ March 2020. These families were approached again on the 18^th^ June 2020, for the same mental health information they had provided prior to the pandemic, together with responses to COVID‐19‐specific questions. By the preregistered date for data collection for this report (4 August), 202 (89%) had responded. Since the sample was drawn from an ongoing population cohort, we were able to assess how representative these 226 families were of those originally recruited (reported in Appendix S1).

### Ethical considerations

Ethical approval for the study was granted by the Cheshire North and West Research Ethics Committee on 27 June 2006 (reference no. 05/Q1506/107), and 7 June 2010 (reference no. 10/H1010/4) and on 22 December 2014 and 8 June 2020 (reference no. 14/NW/1484). All women gave written informed consent at recruitment and at subsequent assessment waves. Child assent was gained at age 11–12.

### Measures

#### Child outcomes

Child depression was assessed using mother and child report on the Short Mood and Feelings Questionnaire (SMFQ; Angold & Stephen, 1995), which includes 13 assessing DSM depression symptoms over the prior 2 weeks. The analysis used a total score and a cut point of ≥12 for child report, the same as that used in the Millennium Cohort Study (Patalay & Fitzsimons, 2018), and shown to be the optimal cut‐off for the identification of clinical depression in adolescents (Thabrew et al., 2018) and ≥11 for parent report (Thapar & McGuffin, 1998).

Child post‐traumatic stress disorder (PTSD) symptoms were assessed using mother and child report on the Child Trauma Scale symptoms scale (Lang & Connell, 2017), which includes six items assessing DSM PTSD symptoms over the prior 30 days. The analysis used a total score and a cut point of ≥6 for both child and parent reports (Lang & Connell, 2018).

Child anxiety symptoms were assessed using mother report on the Short Spence Anxiety Scale (Reardon et al., 2018) which includes eight items assessing anxiety with no defined rating period. The analysis used a total score and a cut point of ≥8.

Child behavioural problems were assessed using the mother report on the Child Behaviour Checklist (CBCL; Achenbach & Rescorla, 2001) Aggressive Behaviour subscale, which includes 18 items assessing disruptive behavioural problems over the prior 6 months. The analysis used a total score and a cut point of ≥65 on the *T* score.

#### Mother outcomes

Maternal depression was assessed using the Patient Health Questionnaire‐9 (PHQ‐9; Kroenke et al., 2001) and maternal anxiety using the Generalised Anxiety Disorder‐7 (GAD‐7; Spitzer et al., 2006). The analysis used a total score and a cut point of ≥10 (Kroenke et al., 2001) and of ≥7 (Plummer et al., 2016), respectively. Psychological relationship abuse over the prior 6 months was assessed by mother report using a short (6‐item) version of the 20‐item Dunedin Relationship Scale (Moffit et al., 1997). High agreement between self‐ and partner reports using this measure has been found. The analysis used a summed score of mother‐ and partner‐perpetrated abuse to index child exposure and partner‐perpetrated abuse only to index mother exposure. A cut point of ≥2 was used to indicate clinically significant abuse.

#### Potential moderating variables

Items were developed to assess the following pandemic‐related variables (questions listed in Appendix S1). Parent in a frontline job (binary variable reflecting 1 = yes for mother or partner, 0 = no to both). Home‐schooling whilst home‐working (binary variable 1 = yes, 0 = no). Financial difficulties during the pandemic (binary variable 1 = 1 or more, 0 = none). Income cut during pandemic (binary 1 = yes 0 = no). Stressful events during the pandemic (binary median split variable = ≥4 stressors).

Maternal prenatal depression was assessed using a cut point of ≥12 on the Edinburgh Postnatal Depression Scale (EPDS; Cox et al., 1987) at 20 weeks gestation. Prior child emotional and behavioural problems were assessed using a *T* score of ≥60 on the mother‐report CBCL internalising and externalising subscales at age 7 years.

Deprivation was assessed using the 2019 IMD (Noble et al., 2019). In this system, postcode areas in England are ranked from most to least deprived based on deprivation in seven domains: income, employment, health, education and training, barriers to housing and services, living environment and crime. Analysis used a binary variable reflecting 1 = most deprived quintile of UK neighbourhoods, 0 = all other quintiles.

### Statistical analysis

The analysis was preregistered (as predicted # 45607; https://aspredicted.org/f8gd8.pdf) prior to the planned cut‐off date of 4^th^ August for receipt/download of follow‐up survey data. Logistic regression was used to identify variables that distinguished responders from non‐responders to the wave pre‐lockdown wave. All total scores, which reflect symptoms, psychological abuse and stressors, showed varying degrees of positive skew. We modelled these using a negative binomial distribution, using the estimated cumulative distributions to estimate the proportion above the accepted thresholds for clinical significance with bootstrapped 1000‐replicate bias‐corrected 95% confidence intervals (CIs). Estimates of relative symptom rates for pre‐ and post‐lockdown scores were obtained using a repeated‐measures generalised linear model set‐up in the Stata procedure gsem (see Appendix S1). This allows for selective loss associated with pre‐lockdown symptom scores and any covariates. Maternal ratings of child symptoms were adjusted for maternal depressive symptoms by including her own contemporaneous depressive symptoms as a covariate with a common pre‐ and post‐lockdown coefficient. Effects of moderators were obtained from the moderator‐defined group, testing whether group differences had changed from their pre‐pandemic level. Since the groups commonly differed pre‐pandemic, the null hypothesis is that the groups changed in proportion, that is, both increasing by the same percentage. Significant moderation implied that the percentage change differed between the groups. As a sensitivity analysis for the effects of cohort attrition from pregnancy, Appendix S1 presents estimates obtained applying inverse probability weights from a logistic model of drop‐out (Appendix S1; Figure S1).

The registered list of moderators was extended by exploratory analyses that examined the differences suggested by recent publications about the pandemic and associated with the known major vulnerabilities. For young adolescents, these were their gender and childhood emotional and behavioural problems; for mothers, their age and previous depression when first recruited, and for both their neighbourhood level of deprivation and exposure to inter‐partner abuse. No other factors were examined. Additionally, a multivariate analysis of variance of the change in child and mother reports of the PTSD items was undertaken to identify the specific profile of change. Analysis was undertaken in Stata v15.1.

## RESULTS

Figure 1 shows the participant flow for the 226 families included in this study, and Table 1 their demographic and pre‐lockdown characteristics. The 202 responders to the post‐lockdown follow‐up (89%) did not differ significantly on any of the measured characteristics from the 226 who provided pre‐lockdown data (Table 1). The majority (66%) of the sample responded within 2 weeks of the survey being sent out, with 90% responding prior to mid‐July when schools closed for the summer. Table S1 gives the descriptive statistics for the study measures at the pre‐ and post‐onset COVID‐19 assessments.

**TABLE 1:** Participant demographic characteristics

	Mean	SD	Range
Time between pre and post questionnaire (*n* = 202)	4.82 months	.95	3–7
Mothers age (*n* = 226)	40.85 years	(5.37)	30–54
Child age (*n* = 226)	11.97 years	(.36)	10–12
		***N***	**%**
Child gender	Male	103/226	45.6
IMD deprivation (2019)	Most deprived quintile	49/221	22.2
Mother ethnicity	White British	219/226	96.9
Mother relationship status	Married or cohabiting	183/226	81
	With a partner who lives elsewhere	19/226	8.4
	Single	26/226	10.6
Mother employment status	Full‐time	92/226	40.7
	Part‐time	110/226	48.7
	Unemployed	5/226	2.2
	Full‐time parent at home	19/226	8.4
Partner employment status	Full‐time	168/226	83.2
	Part‐time	11/226	5.4
	Unemployed	17/226	8.4
	Full‐time parent at home	4/226	2
	Retired	2/226	1
COVID‐19 positive test in parents or child		8/202	4.0

Abbreviations: IMD, Indices of Multiple Deprivation; SD, standard deviation.

### Pandemic impact on young adolescent mental health

**FIGURE 2:**
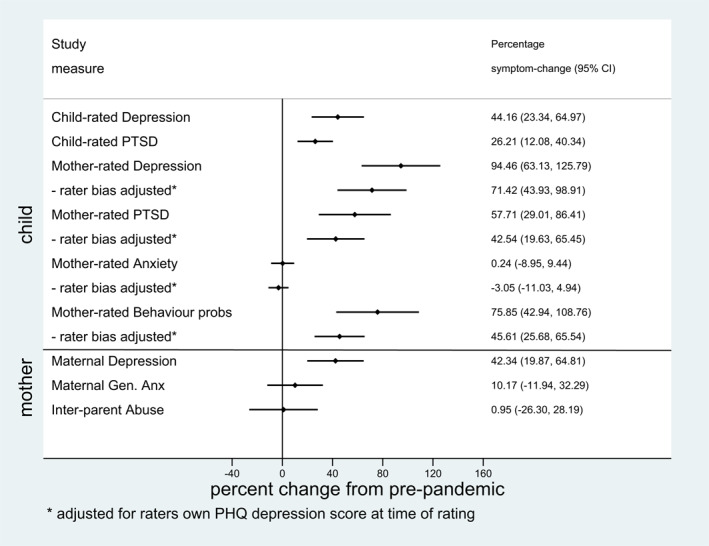
Post versus pre‐pandemic percentage increase in mental health symptoms and behaviour problems, with child symptoms above the horizontal line and maternal symptoms below

Figure 2 shows, above the horizontal line, the percentage changes in symptoms from pre‐lockdown to during lockdown. Children reported a 44% increase in their symptoms of depression (*p* < .001), and a 26% increase in PTSD symptoms (*p* < .001). Based on maternal reports, children's depressive symptoms increased by 94% (*p* < .001); the increase was reduced to 71% (*p* < .001) after adjusting for levels of maternal depression, a potential source of reporting bias. Mothers also reported marked increases in children's PTSD symptoms (58%, *p* < .001) and behaviour problems (76%, *p* < .001); however, they did not report an increase in their anxiety. Sensitivity analyses gave the corresponding estimates of Figure S1 weighted for cohort attrition over the 13 years since recruitment in pregnancy and showed a very similar pattern in both magnitude and statistical significance. Since there was a high correlation between the Child Trauma Screen PTSD questionnaire and the SMFQ (rho = 0.83), we undertook additional exploratory analysis examining the COVID‐19‐related change on the PTSD measure item by item (described further in Appendix S1). This is shown in Figure S2, where it can be seen that the changes were confined to items likely to reflect depression.

#### Pandemic impact on young adolescent mental health in relation to gender and prior childhood emotional and behavioural problems

**FIGURE 3:**
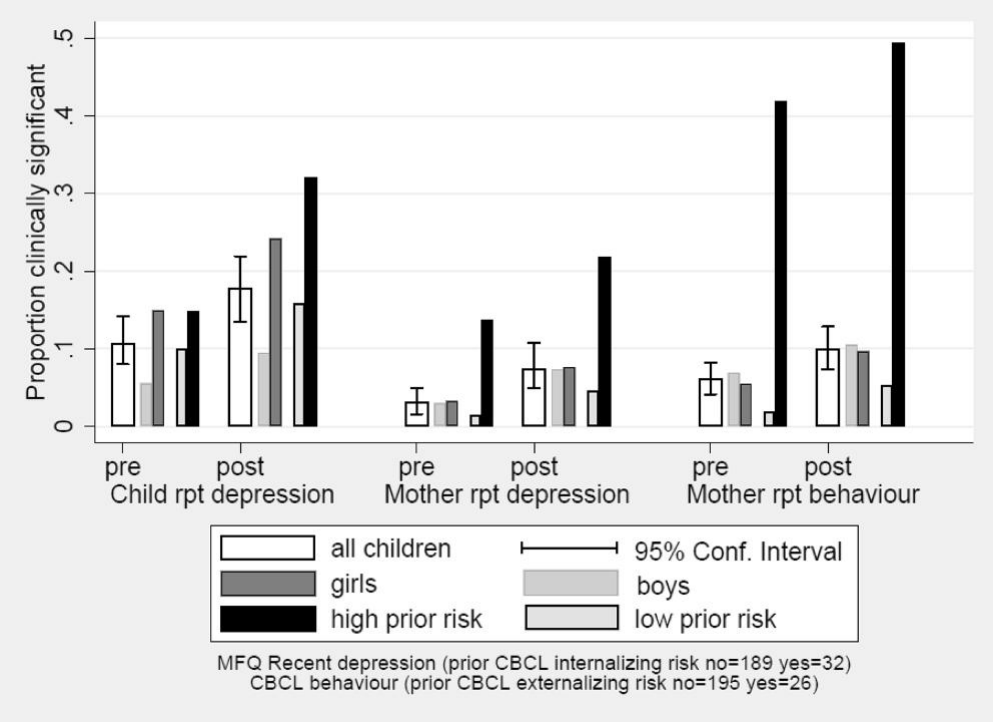
Pre‐ and post‐pandemic prevalence rates of clinically significant child symptoms

Figure 3 shows how COVID‐19 impacted on rates of clinically significant symptoms in relation to two key influences on adolescent depression, gender and pre‐existing emotional and behavioural problems. Based on self‐report, girls had more depressive symptoms than boys' pre‐lockdown (*p* = .001) and both genders experienced a similarly large percentage increase in symptoms during lockdown (interaction term, *p* = .829). Because girls started at a higher level, by mid‐lockdown, the difference was substantial, with almost a quarter of girls having clinically significant symptoms compared to around 10% of boys. By contrast, the maternal reports of child symptoms did not show a gender difference either pre‐ or post‐lockdown. In the case of behaviour problems, this may reflect the within‐sex standardisation of the CBCL T‐score, whereby girls receive a higher *T* score than boys for the same raw score.

Young adolescents' self‐reports of their levels of clinically significant depressive symptoms were also strongly predicted, pre‐lockdown (*p* < .001) and post‐lockdown (*p* < .001), by elevated internalising symptoms 5 years earlier at age 7 years as reported by their mothers. Although the COVID‐related increase was no different proportionally in the low‐ and high‐risk groups (interaction term, *p* = .350), those with prior internalising symptoms had a higher absolute increase, rising to over 30% post‐lockdown. The children's internalising and externalising problems reported by mothers at age 7 years predicted, respectively, both pre‐ and post‐pandemic depression and behaviour problems. For mother‐reported levels of depression, the percentage increase was higher for those without prior symptoms (interaction term *p* = .022). For children reported with lower levels of externalising symptoms at age 7, the percentage increase in mother‐reported behaviour problems was markedly higher (interaction term *p* < .001). This suggests that mothers are reporting a greater COVID‐19‐related proportional increase in symptoms and behaviour problems for many children not previously seen as of concern.

#### Pandemic impact on mothers' mental health

**FIGURE 4:**
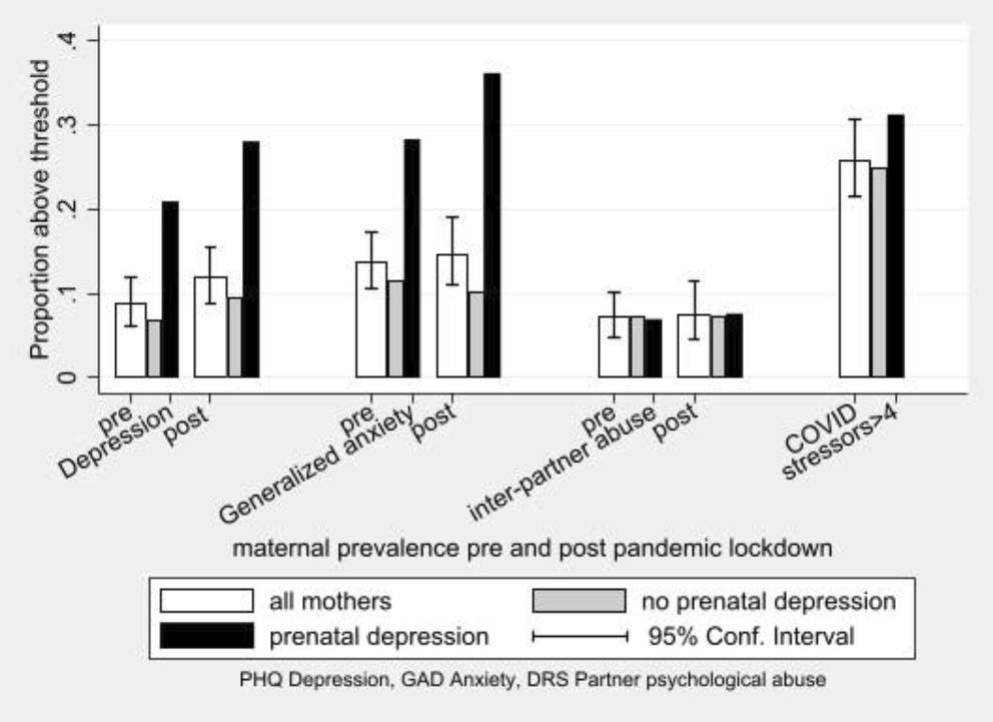
Rates of parent self‐reported clinically significant symptoms

The lower part of Figure 2 shows clinically significant depressive symptoms increasing by 42% in mothers, although there were no clear changes in anxiety or psychological abuse from partners. Figure 4 shows how, similar to adolescent depression, maternal depression was predicted pre‐ (*p* = .004) and post‐lockdown (*p* = .001) by prior symptoms; in this case of clinically significant depression during pregnancy. However, there were no significant difference between those with or without prenatal depression in the proportionate increase in either depression (*p* = .222) or anxiety (*p* = .509). Neither the mothers' exposure to psychological abuse from partner nor their report of the number of COVID stressors differed by prenatal depression status.

#### Moderators of impact on the mental health of children and their mothers

**FIGURE 5:**
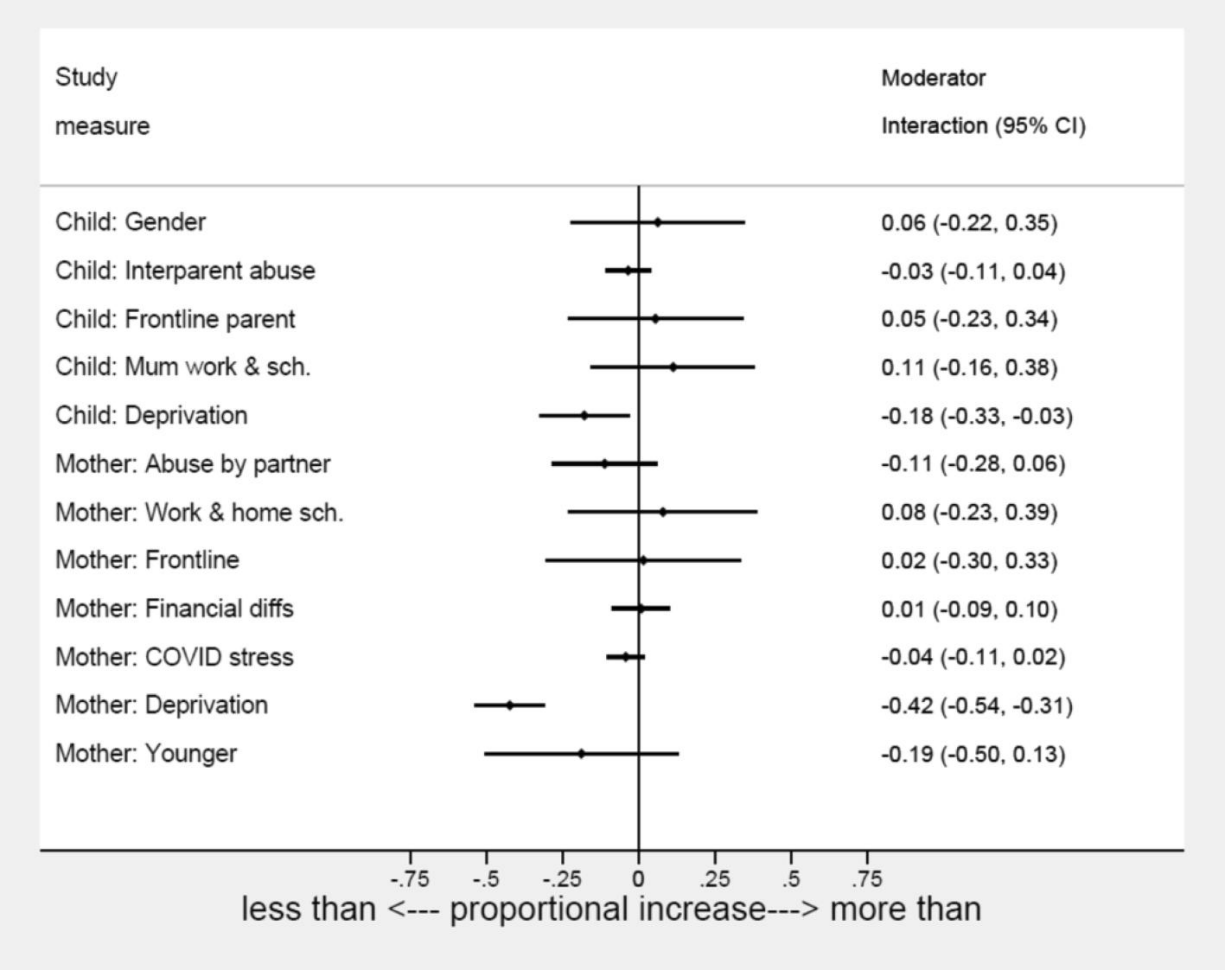
Impact of being in the ‘high‐risk’ category of potential moderators of the effects of the pandemic on the increase in symptoms from pre to post‐pandemic

**FIGURE 6:**
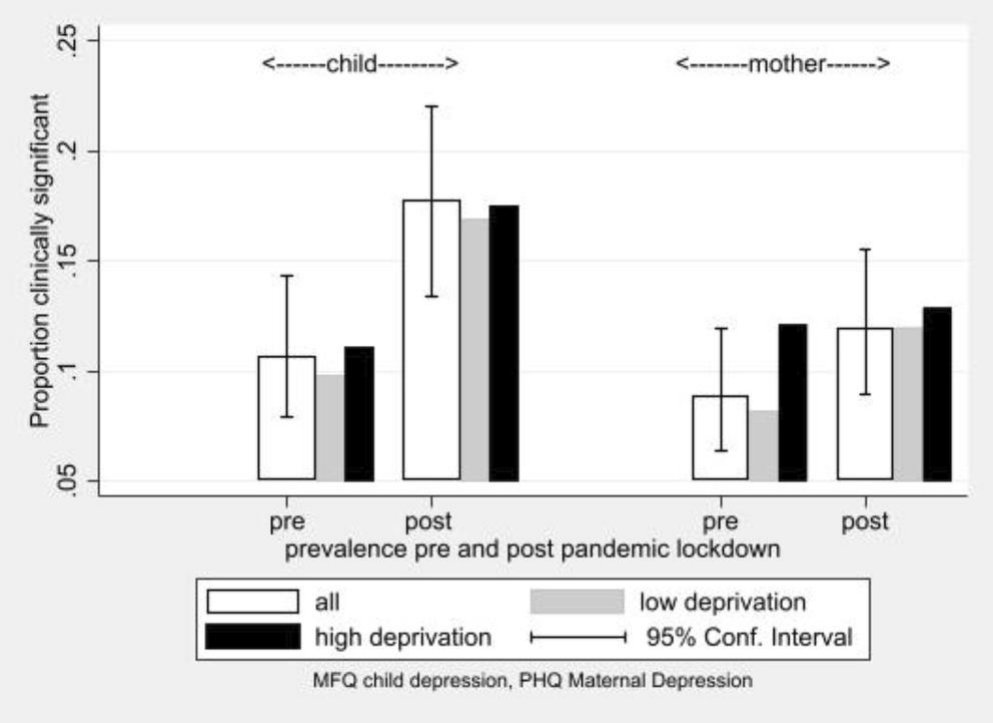
Rates of clinically significant child and mother symptoms of depression comparing those in the most deprived IMD, Indices of Multiple Deprivation Index quintile with the remainder

It can be seen in Figure 5 that of all the moderators of COVID‐19 impact on depression, only deprivation gave 95% CIs that did not cross zero. Figure S3 shows the absolute increase in rates by moderator group, and additional information on the financial moderators is reported in Appendix S1. More marked for depression among mothers than children, in both cases, the proportional rise in symptoms was lower in families in the most deprived UK quintile, than in the less deprived. Figure 6 provides the explanation for this apparent protective effect of deprivation, with higher levels of depression in families in deprived neighbourhoods that changed little after lockdown. By contrast, the prevalence rates increased in the less deprived families, bringing them up towards the level of the deprived.

## DISCUSSION

We assessed the impact of COVID‐19 on child and mother mental health by using repeated measures immediately prior to, and during, lockdown for the pandemic. We find that in the United Kingdom, the pandemic has been associated with a substantial increase in young adolescent mental health problems. According to their own, and their mothers' reports, levels of depression scores in 11–12‐year olds increased by between 44% and 71%, respectively. COVID‐19 exposure widened the gender difference in self‐reported young adolescent depression, absolutely although not proportionately, and added to the risk associated with emotional problems identified when the children were aged 7 years. Mothers reported a 44% increase in their children's disruptive behaviour problems, but not in anxiety levels. Their own symptoms of depression increased by 42% over the same period, but there was no change in their levels of anxiety, and they did not report an increase in psychological abuse in the home. Maternal depression prior to the pandemic was higher among those with prenatal depression 12 years earlier, and rose further post‐lockdown, while neighbourhood deprivation was associated with more pre‐COVID‐19 depression but not with a greater rise post‐lockdown. None of the COVID‐19 associated experiences, such as the presence of a frontline worker in the family, working while home‐schooling, COVID‐19‐related stressful events or financial difficulties, was associated with a disproportionate change in pre‐ to post‐lockdown child or maternal mental health.

A major strength of the study is that we were able to compare the same mental health measures collected during the 3 months prior to the COVID‐19 pandemic and 3 months into the lockdown. The risk of bias associated with poor child or parent mental health was minimised by a high follow‐up pre‐ to post‐lockdown. In this general population birth cohort, we were able to use pregnancy variables to show where attrition over time had not been at random, and account for that in data analyses, thus enhancing the generalisability of our findings. The narrow age range of our sample was both a strength and a weakness. On the one hand, it enabled us to identify an effect at a specific and important developmental period, and on the other hand, it limited the generalisability of our findings. Generalisability is also limited by the lack of ethnic diversity reflecting the demographic characteristics of the Wirral. Whilst the study had good power to detect main effects, statistical power for the moderator effects was the modest. Although we used a measure of PTSD with reported divergent validity from the MFQ in adolescents (Lang & Connell, 2017, 2018), item‐level analysis indicated that the increase we observed may be better accounted for by changes in depression. Finally, the majority of the post‐COVID survey data was collected within a narrow time frame when strict lockdown restrictions were in place (prior to 4 July), but data collection continued until 4 August, meaning that there is some variation in the experiences of the families who responded.

Compared to other studies with pre‐COVID‐19 and post‐lockdown measurement, the finding of an increase in depressive symptoms is consistent with one previous study (Bignardi et al., 2020), but it differs from another which found no change in depressive symptoms in 13–14‐year olds reported by the children (Widnall et al., 2020). The study had a sample size of 770, and so adequate power to detect a small effect; however, it differed from the present study in three key respects. First, the follow‐up rate from pre‐COVID‐19 to post‐lockdown was 44% compared to 89% in this study reducing risk of bias from mood effects. Second, responses were gathered solely online, while in the current study paper collection was also available, reducing the risk of bias associated with lack of access to the Internet. Third, the measure of depression, the Hospital Anxiety and Depression Scale (Zigmond & Snaith, 1983), unlike the SMFQ used in this study, does not provide coverage of DSM symptoms of major depression, and has a predominance of items referring to a loss of interest in usual activities. Our results are consistent with the recent NHS study of a sample of 5–16‐year olds which reported an increase in rates of probable mental disorder reported from 2017 to post‐lockdown (Vizard, Sadler, et al., 2020). Whilst this study benefits from a large sample that allowed examination of multiple potential moderators, similar to Widnall et al. it suffered from a low response rate (45%) and used only online data collection methods. It also differs by reporting only on a broad measure of mental health problems and on a sample with a wide age range spanning childhood and adolescence.

By embedding this study of COVID‐19 impact in a prospective study with recruitment during pregnancy, we were able for the first time to examine how COVID‐19 interacts with prior adjustment. The evidence for young adolescents, according to their own reports of their current depression, was that those who were vulnerable, by virtue of having had elevated emotional symptoms five years earlier, had high higher levels of depression prior to COVID‐19 which were further added to by COVID‐19 exposure. The effect in mothers of prenatal depression 12 years earlier was very similar, underlining the need for evaluated treatments to reduce persistent vulnerability through young adult life. There was no evidence, however, that COVID‐19 had a disproportionate effect in those with previous mental health problems. Furthermore, according to mother reports of young adolescent depression and behaviour problems, there was a greater effect of COVID‐19 among those not previously identified with higher symptoms. Like previous studies that have found a lesser increase in mental health symptoms in the unemployed than the employed (Pierce et al., 2020; Vizard, Davis, et al., 2020), the explanation for lower COVID‐19‐related proportional increase in depression among the most deprived families, appeared to arise from pandemic exposure bringing more of the previously advantaged to similar depression levels as the disadvantaged. It may be that COVID‐19 does not add substantially to the pressures already experienced by those with fewer economic resources, while those with less prior deprivation experience a greater change.

## CONCLUSION

Four implications of our findings give cause for concern and need further investigation. The first arises from what is well established, that two syndromes of emotional and behavioural problems in childhood and adolescence, depression and disruptive behaviour problems, are associated with increased risk for depression later in life, as well as other mental health problems and social difficulties (Clayborne et al., 2019; Copeland et al., 2009; Harrington et al., 1990). Both increased markedly over a short period following the COVID‐19 onset, raising the question of whether COVID‐19 exposure has added risk for adolescent depression that will persist for much longer over the life course. Second, concern for the possible long‐term implications is further raised in children entering adolescence, because this is a period of a rapid increase in depression, when the gender difference is widening and the long‐term vulnerability for depression in women is becoming apparent (Cyranowski et al., 2020). The rise in symptoms during the pandemic brought the prevalence among these 12‐year‐old girls up to the same level (24%) as that reported by 14‐year‐old girls of the Millenium Cohort using the same measure and threshold (Patalay & Fitzsimons, 2018). Third, our findings indicate that referrals for depression and disruptive behaviour problems during the pandemic are likely to comprise both young people with new onsets of mental health problems for which COVID‐19‐related stressors may be the most relevant, and others where COVID‐19 has added to pre‐existing vulnerability with a need for attention to both. Finally, COVID‐19 has added risk for those who have already experienced mental health problems, and may have added disproportionally more for those without previous problems, perhaps creating an additional group of young children at risk for future mental health problems. Only further follow‐up will tell us whether this is the case, or whether COVID‐19 exposure has simply ‘brought forward’ the first episode of depression in children who would have become depressed later in its absence. Meanwhile, the findings underline the need for a better understanding of risk and protective factors for COVID‐19‐related mental health problems as a basis for new treatments.

## CONFLICT OF INTEREST STATEMENT

The author has declared that they have no competing or potential conflicts of interest.

## AUTHORS CONTRIBUTION

Nicola Wright, Jonathan Hill, Helen Sharp & Andrew Pickles conceptualised the study, Nicola Wright & Helen Sharp administered the project, Nicola Wright & Andrew Pickles conducted the analysis, Nicola Wright, Jonathan Hill & Andrew Pickles wrote the original draft and all authors edited the draft.

## ETHICS STATEMENT

Ethical approval for the study was granted by the Cheshire North and West Research Ethics Committee on 27 June 2006 (reference no. 05/Q1506/107), and 7 June 2010 (reference no. 10/H1010/4) and on 22 December 2014 and 8 June 2020 (reference no. 14/NW/1484). All women gave written informed consent at recruitment and at subsequent assessment waves. Child assent was gained at age 11–12. [Corrections made on 22 June 2022, after first online publication: This Ethics Statement has been added in this version.]

## Supporting information

Supplementary Material S1

## Data Availability

Due to ethical constraints supporting data cannot be made openly available. Supporting data are available to bona fide researchers on approval of an application for access. Further information about the data and conditions for access are available at the University of Liverpool Research Data Catalogue: https://doi.org/10.17638/datacat.liverpool.ac.uk/564.
