# Supplementary material for: Interplay between long‐term vulnerability and new risk: Young adolescent and maternal mental health immediately before and during the COVID‐19 pandemic
Source: JCPP Adv. 2021 May 18;1(1):e12008. doi: 10.1111/jcv2.12008 (PMC8206735; doi:10.1111/jcv2.12008)
Supplement: Supplementary file 1 — Supplementary Material S1 [file JCV2-1-e12008-s001.docx]

**Supporting Information – Wright et al.**

**Appendix S1.**

**Comparison of the 226 families analysed to those originally recruited**

The subsample with maternal reports had a lower proportion of mothers aged less than 25 years when recruited in pregnancy (p < .001), somewhat fewer mothers with full-time education beyond age 18 (p = .070) but did not differ on 20 week prenatal depression (p = .298), marital status (single, cohabit, married; p = .648), neighbourhood deprivation quintile (p = .119), smoking during pregnancy (p = .952), obstetric risk (p = .924), birthweight adjusted for gestation (p = .326) nor child gender (p = .187). For the 187 child reports included the pattern was the same with the exception of neighbourhood deprivation which was associated with poorer response (p = .050).

**Pandemic related questions used in the study**

*Parent in a front-line job*: Mothers answered the following questions about themselves or their partner living in their home “Do you feel your work places you at higher risk of contracting COVID-19? (e.g., medic, nurse, healthcare assistant etc)” and “Does your work involve caring for or working near people who are known to have the COVID-19 virus? (e.g., NHS setting or care home)” (yes or no response)

*Home-schooling whilst home-working:* Mothers answered “Are you expected to work from home whilst caring for your children at home?” (yes or no response).

*Financial difficulties during the pandemic:* mothers answered six questions assessing use of food banks, being unable to pay essential bills, being unable to pay rent/mortgage, being unable to pay for food, increasing debt to cover essential costs and being at risk of losing their home (yes or no response)

*Income cut during pandemic:* mothers were asked “During the COVID-19 pandemic has your household income been cut?” and if “yes”, they reported how much on a 9-point scale from 10%-90%..

COVID-19 related stressful events: mothers answered “yes or no” to 24 events:

1. Your first born child not being able to see one of their parents that they would normally have regular contact with

2. Needing to take supplies to a relative/friend who is self-isolating (NOTE: Self-isolating means staying at home and avoiding contact with people outside their household)

3. Having to sort out different childcare for your children

4. Having to cancel a holiday

5. Having to cancel another important social event

6. Having to cancel a house sale/buy

7. Having to stop an educational course you were completing

8. Being separated from your partner

9. Being separated from one or more of your children

10. An ex-partner limiting your normal contact with your child(ren)

11. Being separated from a friend or family member you are very close to

12. Having to stop treatment for a physical problem (e.g. physiotherapy) or not being able to have treatment you have been waiting for (e.g. minor surgery)

13. Having to stop treatment for a psychological problem (e.g. therapy, AA groups)

14. You are pregnant and your antenatal care is affected

15. Not being able to care for elderly relatives in the usual way

16. Looking after a person with special needs, disabilities or a vulnerable adult at home without usual support

17. Lack of public transport to get to the shops for supplies or to work

18. Difficulty getting medicines

19. Difficulty getting essential supplies (e.g. food and cleaning products)

20. Live in a place without a garden or in a high rise flat

21. Overcrowded living situation

22. Being in the same house on lockdown with a family member you do not get on well with

23 Had difficulty keeping your children occupied and healthy

24. Not being able to get through to doctors or 111

**Table S1.** Descriptive statistics for the study measures at the pre- and post-onset COVID-19 assessments.

|  | **Possible range** | **Pre-COVID assessment** | | | **Post-COVID assessment** | | |
| --- | --- | --- | --- | --- | --- | --- | --- |
| **Study measure** |  | N | Mean | SD | N | Mean | SD |
| Child-rated depression | 0-26 | 187 | 4.80 | 5.15 | 164 | 6.59 | 6.00 |
| Child rated PTSD | 0-24 | 187 | 3.89 | 4.07 | 164 | 4.81 | 4.50 |
| Mother-rated child depression | 0-26 | 226 | 2.00 | 3.33 | 202 | 3.74 | 4.24 |
| Mother-rated child PTSD | 0-24 | 226 | 1.41 | 2.28 | 202 | 2.24 | 2.80 |
| Mother-rated child anxiety | 0-24 | 226 | 4.43 | 3.30 | 202 | 4.43 | 3.44 |
| Mother-rated beh. problems total score | 0-38 | 226 | 3.59 | 4.90 | 202 | 5.23 | 5.28 |
| Mother-rated beh. problems T score | 50-100 | 226 | 53.19 | 6.22 | 202 | 55.11 | 6.82 |
| Maternal depression | 0-27 | 226 | 3.31 | 4.46 | 202 | 4.59 | 4.10 |
| Maternal anxiety | 0-21 | 225 | 2.89 | 3.88 | 202 | 3.16 | 3.61 |
| Inter-parent abuse^1^ | 0-6 | 198 | .59 | 1.27 | 177 | .59 | 1.34 |
| COVID-19 stressors | 0-24 | - | - | - | 202 | 4.47 | 2.07 |

^1^Inter-parent abuse only applicable for mothers with partners

**Model Based Estimates**

Prevalence estimates were obtained from the fitting of negative binomial regression (Stata command nbreg) and calculating the cumulative predicted probability for symptom counts up to the clinical threshold (using the Stata utility prcounts). Confidence intervals were calculated from the estimate distribution from 1000 bootstrap samples.

Estimates of change were obtained from a repeated measures version of the negative binomial regression that accounted for the correlation over time by means of a normally distributed random intercept (Stata command gsem). A common shape parameter for pre and post assessments was used unless a test of inequality was significant. With a log link-function the estimate of change was exponentiated to provide a post *versus* pre rate-ratio, expressed as a percentage increase over the pre-pandemic symptom level. Estimated by maximum likelihood these models assumed that sample attrition between the pre and post lockdown surveys was missing at random.

All models were checked against models assuming a Poisson conditional error distribution and robust standard errors. Where conditional errors showed no overdispersion (and hence the negative binomial shape parameter tended towards minus infinite) estimates of relative symptom rates (transformed to percentage increase/decrease) and confidence intervals were taken from the Poisson model.

**Attrition weighted estimates**

Much of the difference in sample size from the original cohort arose because only the families with the earlier birth dates had been approached before lockdown. As reported in the main paper, we also found, with the exception of maternal age, very little difference on a list of variables likely to be associated with attrition between those included in the analysis of this paper and the original cohort. Nonetheless we examined the potential impact of attrition bias by estimating inverse probability weights from a logistic regression for the whole cohort, including those who had not been approached to participate in the pre-pandemic phase. The variables from the original recruitment wave included as likely predictors of participation were maternal age, maternal education, maternal smoking, maternal depression, IMD deprivation, marital status and the sex of the child. The confidence intervals for weighted estimates of the percentage increase in symptom levels were obtained from the corresponding intervals for the rate-ratio estimates assuming symptoms to be negative binomial distributed and a sandwich/robust estimator of the parameter covariance matrix (Binder, 1983).

Binder, D. A. 1983. On the variances of asymptotically normal estimators from complex surveys. International

Statistical Review 51: 279–292.

**Figure S1**. Post versus pre pandemic percentage increase in mental health symptoms and behaviour problems with analysed sample weighted to match the characteristics of the 1233 mothers and babies of the original cohort.

**Pre-post Change for individual Items of the Child Trauma Scale**

Four of the six items on the CTS are also characteristic of depression (“trouble feeling happy”, “trouble sleeping”, “hard to concentrate or pay attention”, “feel alone and not close to people around you”). The plot below shows the simple difference in item scores between pre and post pandemic assessments. Wald tests from a multivariate regression of the 12 item (6 mother and 6 child report) change-scores showed a highly significant difference for the mean change for the 8 depression related items as compared to the mean change of the 4 PTSD specific items (p<.001) which showed no evidence of mean change (p=.610).

**Figure S2.** Change in 6 item scores of the child and mother report of the Child Trauma Scale between pre and post-pandemic assessments.

Effects of possible moderators of change in depression of mothers and children.

We pre-registered two financial related variables, COVID-19 financial stressors and an income cut during the pandemic. Given space limitations and similarity of findings we display only the financial difficulties stressor (income cut moderation gave p=.924 for child MFQ and p=.690 for mother PHQ)

**Figure S3.** Change in rates of clinically significant depression (MFQ for children, PHQ for mothers) with 95% bootstrap confidence intervals for children and parents grouped by potential moderators. Labels show the frequencies of participants by group included in each analysis.
